# The methyltransferase domain of dengue virus protein NS5 ensures efficient RNA synthesis initiation and elongation by the polymerase domain

**DOI:** 10.1093/nar/gku666

**Published:** 2014-09-10

**Authors:** Supanee Potisopon, Stéphane Priet, Axelle Collet, Etienne Decroly, Bruno Canard, Barbara Selisko

**Affiliations:** 1 Aix-Marseille Université, AFMB UMR 7257, 13288 Marseille, France; 2 CNRS, AFMB UMR 7257, 13288 Marseille, France

## Abstract

Viral RNA-dependent RNA polymerases (RdRps) responsible for the replication of single-strand RNA virus genomes exert their function in the context of complex replication machineries. Within these replication complexes the polymerase activity is often highly regulated by RNA elements, proteins or other domains of multi-domain polymerases. Here, we present data of the influence of the methyltransferase domain (NS5-MTase) of dengue virus (DENV) protein NS5 on the RdRp activity of the polymerase domain (NS5-Pol). The steady-state polymerase activities of DENV-2 recombinant NS5 and NS5-Pol are compared using different biochemical assays allowing the dissection of the *de novo* initiation, transition and elongation steps of RNA synthesis. We show that NS5-MTase ensures efficient RdRp activity by stimulating the *de novo* initiation and the elongation phase. This stimulation is related to a higher affinity of NS5 toward the single-strand RNA template indicating NS5-MTase either completes a high-affinity RNA binding site and/or promotes the correct formation of the template tunnel. Furthermore, the NS5-MTase increases the affinity of the priming nucleotide ATP upon *de novo* initiation and causes a higher catalytic efficiency of the polymerase upon elongation. The complex stimulation pattern is discussed under the perspective that NS5 adopts several conformations during RNA synthesis.

## INTRODUCTION

Viral RNA-dependent RNA polymerases (RdRps) responsible for the replication and transcription of single-strand RNA virus genomes are of utmost interest as targets in the development of antiviral drugs. RdRps exert their function in the context of replication/transcription complexes consisting of viral and often also host proteins (1,2). Within these complexes the polymerase activity is highly regulated by RNA elements and/or other proteins. Regulation of viral RdRp activities by other viral proteins or domains within multi-domain polymerases has, for example been demonstrated for positive-strand RNA viruses such as hepatitis C virus (HCV) (3), classical swine fever virus (CSFV) (4), SARS-coronavirus (Subissi, L., Posthuma, C.C., Collet, A., Zevenhoven-Dobbe, J.C., Gorbalenya, A.E., Decroly, E., Snijder, E.J., Canard, B. and Imbert, I. (2014) One severe acute respiratory syndrome coronavirus protein complex integrates processive RNA polymerase and exonuclease activities. Proc. Natl. Acad. Sci. U.S.A., submitted) and for negative-strand RNA viruses (5,6). Here we investigate the regulation of the RdRp activity of the polymerase domain of protein NS5 of dengue virus (DENV), by the NS5 methyltransferase domain. Understanding the interplay between proteins or their domains within the DENV replication/transcription complex will provide a possibility to interfere with protein–protein interaction, a strategy which gains attractiveness among antiviral drug developers (7).

DENV belongs to the *Flavivirus* genus, which comprises around 50 virus species (8). Besides DENV the genus contains other important human pathogens such as yellow fever virus (YFV), West Nile virus (WNV), Japanese encephalitis virus (JEV) and tick-borne encephalitis virus (TBEV). The *Flavivirus* genus is part of the *Flaviviridae* family that contains two other genera: *Hepacivirus* (e.g. HCV) and *Pestivirus* (e.g. Classical swine fever virus (CSFV), bovine viral diarrhea virus (BVDV)) (9). DENV is currently endemic in tropical and subtropical countries around the world (10) and its impact on global health is rising. According to a recent estimation about 390 million people are infected annually by DENV worldwide, of which around 100 million people develop clinical dengue with any level of disease severity (11). DENV causes dengue fever, which can develop into hemorrhagic fever and dengue shock syndrome. DENV exists in five serotypes (DENV-1 to 5) (12). Despite its large burden to human health, neither vaccine nor a specific antiviral drug is currently available against this virus.

The DENV RNA genome is made of about 11 000 nucleotides. It bears a type I RNA cap (m^7^GpppAmG) at its 5′-end and has no poly A tail at its 3′-end. The genome codes for three structural proteins (capsid, membrane, envelope protein) and for seven non-structural proteins (NS1, NS2A, NS2B, NS3, NS4A, NS4B, NS5) (8). NS5 is the largest (104 kDa) and most conserved non-structural protein within the DENV serotypes and the *Flavivirus* genus. During the viral lifecycle NS5 is associated with other viral and cellular proteins within the viral replication complex localized in the perinuclear endoplasmic reticulum of the infected cell (13). This complex fulfills multiple functions related to genome replication/transcription and capping. NS5 carries several essential enzymatic activities hosted in two domains: (i) the N-terminal methyltransferase domain (NS5-MTase, residues 1–263, 30 kDa) and (ii) the C-terminal RdRp domain (NS5-Pol, residues 272–900, 74 kDa). Both domains are connected by a flexible linker (14). The NS5-MTase domain (reviewed in (15)) catalyzes RNA cap methylation at both the N7 position of the cap guanosine and the 2′O position of the first nucleotide of the neo-synthesized positive-strand RNA. It might also harbor the DENV guanylyltransferase activity (16).

The NS5-Pol domain is responsible for the replication/transcription of the viral genome. It is able to initiate the synthesis of new viral RNA in the absence of a primer in a process named *de novo* RNA synthesis (17,18). Much like the related HCV RdRp (19), NS5-Pol synthesizes RNA in three main phases: *de novo* initiation (i.e. primer synthesis), transition and elongation. It has been shown *in vitro* that, first, NS5-Pol specifically generates short primers (mainly dimers pppAG but also trimers) over the 3′-end of RNA templates ending in 5′-CU-3′, which corresponds to the strictly conserved 3′-end of *Flavivirus* genomes and antigenomes (20). Then the protein undergoes a structural/mechanistic transition from a *de novo* initiation to an elongation conformation before it continues RNA synthesis in a much faster and processive elongation mode. The 5′-end of the neo-synthesized genomic strand is decorated with an RNA cap. The NS5-MTase domain is involved RNA cap formation, whose precise timing remains unknown. Being part of NS5, the NS5-MTase domain may influence the different steps of RNA synthesis promoted by the NS5-Pol domain. Some evidence exists for the interaction between the two domains but little is known on a possible inter-regulation of their respective enzymatic activities. Genetic interaction between NS5-MTase and NS5-Pol has been reported for DENV (21) and WNV (22). In addition, the interaction between recombinant WNV NS5-MTase and NS5-Pol was demonstrated *in vitro* (23). The NS5-Pol domain was shown to enhance the internal RNA methylation activity of the NS5-MTase domain in the context of the full-length NS5 proteins of DENV and WNV (24). The influence of the NS5-MTase domain on NS5-Pol activity was tested comparing RdRp activities of recombinant NS5 and NS5-Pol (18,25,26) but contradictory results were reported depending on assay conditions. The activity of DENV NS5 has been shown to be 20-fold higher than that of NS5-Pol in the presence of catalytic Mg^2+^ ions but similar in the presence of Mn^2+^ ions (25,26). We found that the *K_M_* values of GTP during poly(rG) synthesis for DENV-2 and WNV NS5 in the presence of Mn^2+^ were 2-fold lower than that of their corresponding NS5-Pol domains (18). Thus, the question of a possible stimulation of the *Flavivirus* NS5-Pol activity by NS5-MTase remains to be further clarified, and especially the influence of NS5-MTase domain during the initiation, transition and/or elongation steps of RNA synthesis catalyzed by NS5-Pol.

The structure of the NS5-Pol domains of several flaviviruses (DENV (27), WNV (21) and JEV (28)) have been determined. As other viral RdRps, the *Flavivirus* NS5-Pol domain adopts a so-called right hand structure, which contains three subdomains: fingers, thumb and palm. The completely encircled active site is found at the center of the molecule at the end of the RNA template and NTP entry tunnels. Typical for an RdRp initiating RNA synthesis *de novo*, *Flavivirus* NS5-Pol domains contain a structural priming element, a loop protruding from the thumb subdomain toward the active site. The priming loop of DENV NS5-Pol has been shown to be essential for *de novo* initiation (20). Although these general features of NS5-Pol have been deciphered, the existing structures do not correspond to an active conformation of the protein since the RNA entry and NTP tunnels are not completely formed (reviewed in (29)). Interestingly, JEV NS5-Pol in complex with GTP or ATP adopts a potentially active state, which could be close to an initiation state (28). There is no structure of NS5-Pol corresponding to an elongation conformation. Initiation and elongation conformations are expected to be considerably different, as it has been shown for HCV RdRp (reviewed in (29)). The *de novo* initiation conformation should be completely closed with the priming element in place to position the first (priming) and second nucleotide in front of the 3′-end of the template. During the subsequent conformational change toward the elongation, the priming element has to move away from the active site; and the polymerase should adopt a more open conformation to harbor and then allow egress of the nascent double-strand RNA. It is possible that the interactions/interfaces between the NS5-MTase and NS5-Pol domains change during the change from one conformation of NS5-Pol to the other. NS5 would thus adopt multiple conformations. This latter hypothesis is supported by SAXS measurements that detected a variety of extended conformations and a small subset of compact conformations in solution (30). The crystal structure of one of these compact forms has recently been solved for NS5 of JEV (14). The structure revealed a hydrophobic interaction zone where the NS5-MTase docks to the NS5-Pol between the RNA and the NTP entry tunnel. The authors suggested that the flexible linker, whose trace is not resolved in the structure, would allow the NS5-MTase domain to be placed in different positions from that captured in the JEV NS5 structure (14).

In this study, we investigated whether the methyltransferase domain, NS5-MTase, of protein NS5 of DENV regulates the RdRp activity of the polymerase domain NS5-Pol. For this, we compared the steady-state polymerase activities of DENV-2 recombinant NS5 and NS5-Pol using different biochemical assays allowing the dissection of the initiation, transition and elongation steps. We show here that NS5-MTase indeed regulates the RdRp activity by stimulating both the *de novo* initiation and the elongation phase of RNA synthesis catalyzed by NS5-Pol. We show that this stimulation is related to a higher affinity of NS5 toward the single-strand RNA template, toward the priming ATP upon *de novo* initiation, and a higher catalytic efficiency upon RNA synthesis elongation. Our results propose that NS5-MTase may interact in a different manner with the NS5-Pol domain during the initiation and elongation steps of RNA synthesis.

## MATERIALS AND METHODS

### Nucleic acids

Dengue antigenomic RNA template of 10 nt (T_10_: 5′-ACUAACAACU-3′) was chemically synthesized by Dharmacon. Dengue antigenomic RNA template of 20 nt (T_20_: 5′-GUCCACGUAGACUAACAACU-3′) and RNA primer of 10 nt (P_10_: 5′-AGUUGUUAGU-3′) either bearing a hydroxyl group or a 6-carboxyfluorescein group (6-FAM) at the 5′-end were chemically synthesized by Biomers.net. Homopolymeric RNA templates poly(rC) and poly(rU) were obtained from GE Healthcare. The DENV minigenome was produced by *in vitro* transcription as described in (18).

### Protein expression and purification

#### NS5 and NS5-Pol

The genes coding for N-terminal His_6_-tagged NS5 and NS5-Pol (serotype 2, strain New Guinea C) as defined in (18) were cloned in a pQE30 expression plasmid. Proteins were produced in *Escherichia coli* (*E. coli*) NEB Express (New England Biolabs) cells transformed with the pRare2LacI helper plasmid (Novagen). Cells were grown in Luria broth until the OD_600_ value reached 0.6. Protein expressions were then induced by 50 μM IPTG after addition of EtOH to a final concentration of 2% and a cold shock (2 h at 4°C) and then carried out overnight at 17°C. After centrifugation cell pellets from 2 l bacterial culture were resuspended in 50 ml lysis buffer (50 mM sodium phosphate pH 7.5, 500 mM NaCl, 20% glycerol, 0.8% Igepal, 22 μg/ml DNase I, 0.2 mM benzamidine, 5 mM β-mercapthoethanol and 1 mg/ml lysozyme). After 40 min of incubation at 4°C, cell lysate was sonicated and then centrifuged. The supernatant was incubated in batch with 3 ml TALON metal-affinity resin slurry (Clontech) for 1.5 h at 4°C. Beads were washed once with washing buffer (50 mM sodium phosphate pH 7.5, 20% glycerol, 0.8% Igepal, 5 mM β-mercapthoethanol, 1 M NaCl and 10 mM imidazole) and once with washing buffer without Igepal. The proteins were eluted with washing buffer containing 500 mM NaCl, 250 mM imidazole and 250 mM glycine. In the case of NS5, size exclusion chromatography (SEC) was used as a second purification step using a Superdex 200 HR 16/20 column (GE Healthcare) and SEC buffer (HEPES pH 7.5, 300 mM NaCl, 10% glycerol, 1 mM DTT). In the case of NS5-Pol, heparin affinity purification was used as a second purification step using a HiTrap heparin column (GE Healthcare) and heparin affinity buffer (HEPES pH 7.5, a gradient from 150 mM to 1 M NaCl, 20% glycerol, 1 mM DTT). NS5 and NS5-Pol were then concentrated up to around 8 mg/ml (78 μM NS5, 108 μM NS5-Pol) and stored at -20°C after a final dialysis into 20 mM HEPES pH 7.5, 300 mM NaCl, 40% glycerol and 1 mM DTT. Protein purity was higher than 95% as judged by SDS-PAGE.

#### NS5-MTase

The gene coding for N-terminal His_6_-tagged NS5-MTase (serotype 2, strain New Guinea C, residues 1–269) was cloned in a Gateway^®^pDEST^TM^14 expression plasmid. NS5-MTase was produced in Shuffle^®^ *E. coli* Express (New England Biolabs). Cells were cultured in 2YT broth until an OD_600_ value of 0.6 was reached. Protein expression was induced by 100 μM IPTG and a cold shock (4 h at 4°C) and then carried out overnight at 17°C. After centrifugation cell pellets from 1 l bacterial culture were resuspended in 30 ml lysis buffer (50 mM HEPES pH 7.5, 300 mM NaCl, 10 mM imidazole, 5 mM MgSO_4_, 0.5 mM DTT, 1 mM PMSF, 10 μg/ml DNase I, 0.5% Triton, 0.25 mg/ml lysozyme). After 30 min of incubation at 4°C, cell lysate was sonicated and then centrifuged. The supernatant was incubated in batch with 1 ml TALON metal-affinity resin slurry (Clontech) for 30 min at 4°C. Beads were washed three times with washing buffer (50 mM HEPES pH 7.5, 20 mM imidazole, 300 mM NaCl, 5 mM MgSO_4_ and 0.5 mM DTT). The protein was eluted with washing buffer containing 250 mM imidazole. NS5-MTase was concentrated up to around 9 mg/ml (350 μM) and stored at -20°C after a final dialysis into 20 mM HEPES pH 7.5, 300 mM NaCl, 40% glycerol and 1 mM DTT. Protein purity was higher than 95% as judged by SDS-PAGE.

### Determination of melting temperature (*T_m_*) values

*T_m_* values of NS5 and NS5-Pol were determined by a thermo-fluor based assay as described in (20). Briefly, 16.1 μl of elongation buffer 50 mM sodium glutamate buffer, pH 7.5 containing 50 mM arginine, 5 mM DTT, 5 mM MgCl_2_ and 15% glycerol were mixed with 5.4 μl protein at a concentration of 2 mg/ml in protein storage buffer containing 40% glycerol. Final protein concentrations were 0.5 mg/ml, which corresponds to molar concentrations of 4.8 μM of NS5 and 6.8 μM of NS5-Pol. We had tested before for NS5 that within this molar concentration range the *T_m_* value remained constant. Finally, 3.5 μl Sypro Orange was added; and thermal denaturation was followed by measuring fluorescence emission. Denaturation midpoints of NS5 and NS5-Pol were calculated using the Boltzmann equation in GraphPad Prism.

### Determination of chemical denaturation midpoints (guanidine hydrochloride)

Denaturation mixtures (150 μl) were prepared in wells of a black 96-well plate (Greiner bio-one, Cat.No 655096) containing 50 mM sodium glutamate buffer, pH 7.5 with 5 mM DTT, 5 mM MgCl_2_, 15% glycerol, increasing concentrations of guanidine hydrochloride (0 to 5 M) and protein at final concentrations of 0.5 mg/ml (NS5) and 1.1 mg/ml (NS5-Pol). Protein was added last. NS5 and NS5-Pol denaturation was followed by measuring fluorescence emission at 300–460 nm (excitation at 280 nm). Denaturation midpoints of NS5 and NS5-Pol were calculated by the Boltzmann equation using the GraphPad Prism software as in (20).

### Fluorescence polarization test

Fluorescence polarization mixtures (100 μl) were prepared in wells of a black 96-well plate (Corning, Cat.No 3686) containing T_20_ buffer (50 mM HEPES pH 8.0, 10 mM KCl, 10 mM DTT, 5 mM MgCl_2_) with 10 nM T_20_ or P_10_/T_20_ buffer (50 mM sodium glutamate pH 7.5 containing 50 mM arginine, 5 mM DTT, 5 mM MgCl_2_, 30 mM NaCl, 5% glycerol) with 8 nM P_10_/T_20_, and increasing concentrations of NS5 (7 nM–4 μM), NS5-Pol (0.1–5 μM) and NS5-MTase (0.1–8 μM). The proteins were added last. Fluorescence polarization values were measured after 10 min at 520 nm (excitation 485 nm). The dissociation constant (*K_D_*) values were calculated using the specific one-site binding equation with Hill slope (*h*) = 1, (*Y* = *B*_max_ *X^h^/(*K_d_^h^* + *X^h^*)) using the GraphPad Prism software: *Y* = polarization values, *B*_max_ = polarization maximum, *X* = protein concentration.

### RdRp assay 1: on homopolymeric and heteropolymeric templates analyzed by filter binding

Reactions were done in HEPES reaction buffer (50 mM HEPES pH 8.0, 10 mM KCl, 10 mM DTT) containing 2 mM MnCl_2_, 5 mM MgCl_2_, with (i) 100 nM poly(rC), 40 nM protein, 20 μM GTP and 0.02 μCi/μl [^3^H-GTP] (Perkin-Elmer) or (ii) 100 nM poly(rU), 100 nM protein, 200 μM ATP, 0.02 μCi/μl [^3^H-ATP] (Perkin-Elmer) or (iii) 100 nM DENV minigenome, 100 nM protein, 300 μM ATP, GTP, CTP, 4 μM UTP, 0.04 μCi/μl [^3^H-UTP] (Perkin-Elmer). Reactions were started by adding a premix of divalent ion (Mn^2+^ or Mg^2+^), NTPs, KCl and HEPES buffer and incubated at 30°C. Samples of 10 μl were taken at given time points and added to 30 μl 100 mM EDTA pH 8.0. The reactions were passed onto DEAE filtermats (Perkin-Elmer); and then filters were washed three times with 0.3 M ammonium formate pH 8.0 in case of poly(rC) and minigenome, but with 0.2 M ammonium formate in case of poly(rU), twice with H_2_O, once with EtOH and dried. Liquid scintillation fluid was added and incorporation was measured in counts per minute (cpm) by using a Wallac MicroBeta TriLux Liquid Scintillation Counter.

### RdRp assay 2: on DENV minigenome template analyzed by formaldehyde-agarose gels

Reactions were done at 30°C in HEPES reaction buffer (see RdRp assay 1) containing 100 nM minigenome, 200 nM protein, 500 μM GTP, 500 μM CTP, 4 μM UTP with [α-^32^P]-UTP at 0.4 μCi/μl in the presence of 5 mM MgCl_2_ or 0.1 μCi/μl in the presence of 2 mM MnCl_2_ and 100 or 500 μM ATP as indicated in Figure 2. Reactions were started by adding a premix of divalent ion (Mn^2+^ or Mg^2+^), CTP, UTP, KCl and HEPES buffer and 10 μl samples were taken at given time points. Samples were added to 1 μl 100 mM EDTA pH 8.0 and then completed with 13.7 μl sample mix (40 mM MOPS buffer, pH 7.0 containing 10 mM sodium acetate, 85 mM EDTA, 2 M formaldehyde, 83.3% formamide), and 1 μl loading buffer after a 10 min incubation at 70°C. The reaction products were analyzed on formaldehyde-agarose gels. RNA product bands were visualized using photo-stimulated plates and the fluorescent Image Analyzer FLA3000 (Fuji) and quantified using Image Gauge (Fuji).

**Figure 1.**
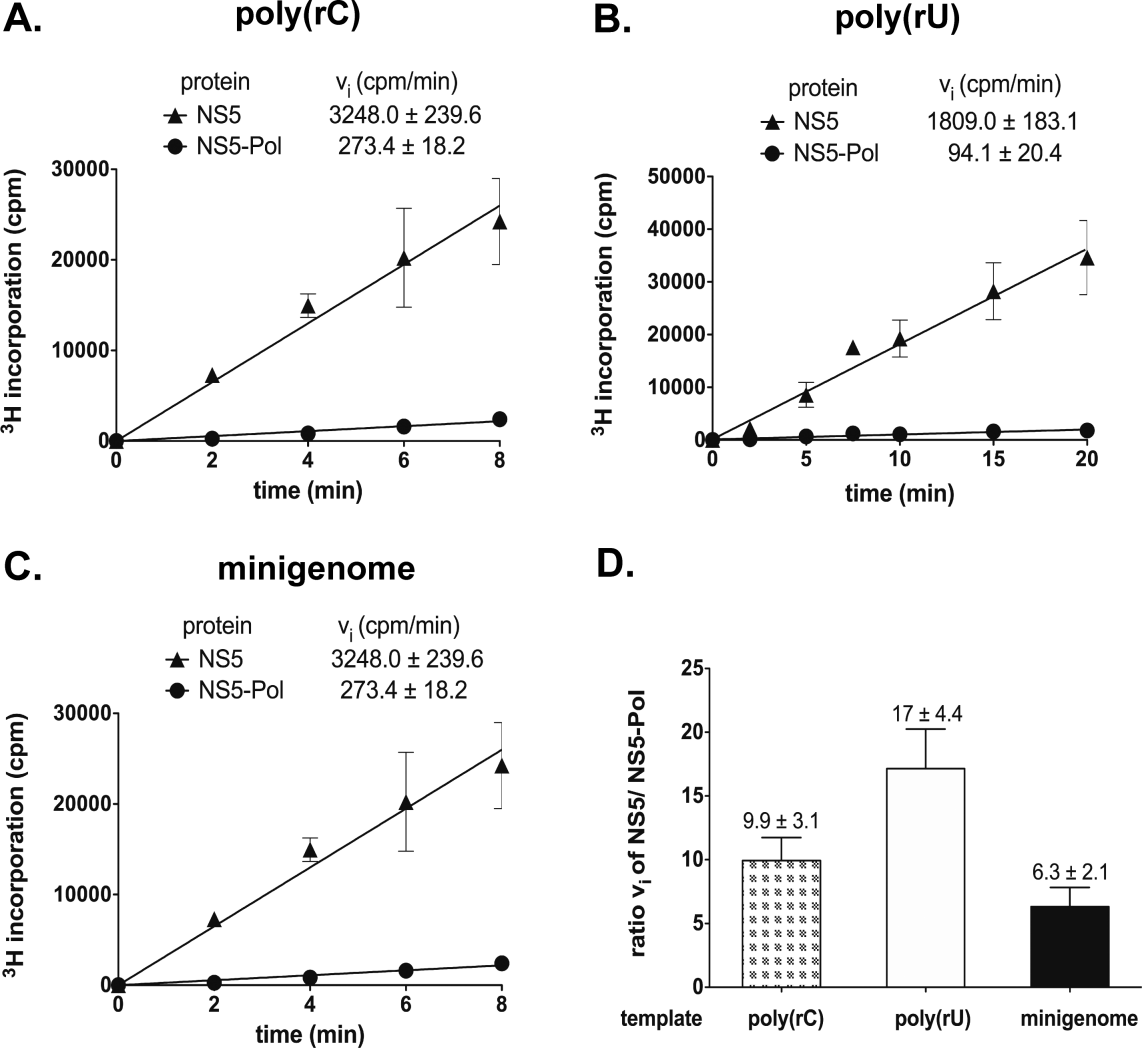
NS5 and NS5-Pol activity on homopolymeric and heteropolymeric templates analyzed by filter binding. Reactions kinetics were determined as given in Materials and Methods (RdRp assay 1). Reactions were done in HEPES reaction buffer with (**A**) 100 nM poly(rC), 40 nM protein, 20 μM GTP, (**B**) 100 nM poly(rU), 100 nM protein, 200 μM ATP, (**C**) 100 nM minigenome, 100 nM protein, 300 μM ATP, CTP, GTP, 4 μM UTP. Incorporation of ^3^H-labeled NTPs was measured in counts per minute (cpm). (**D**) Ratios of NS5 and NS5-Pol activities are given for different RNA templates. The tests were done in triplicates.

**Figure 2.**
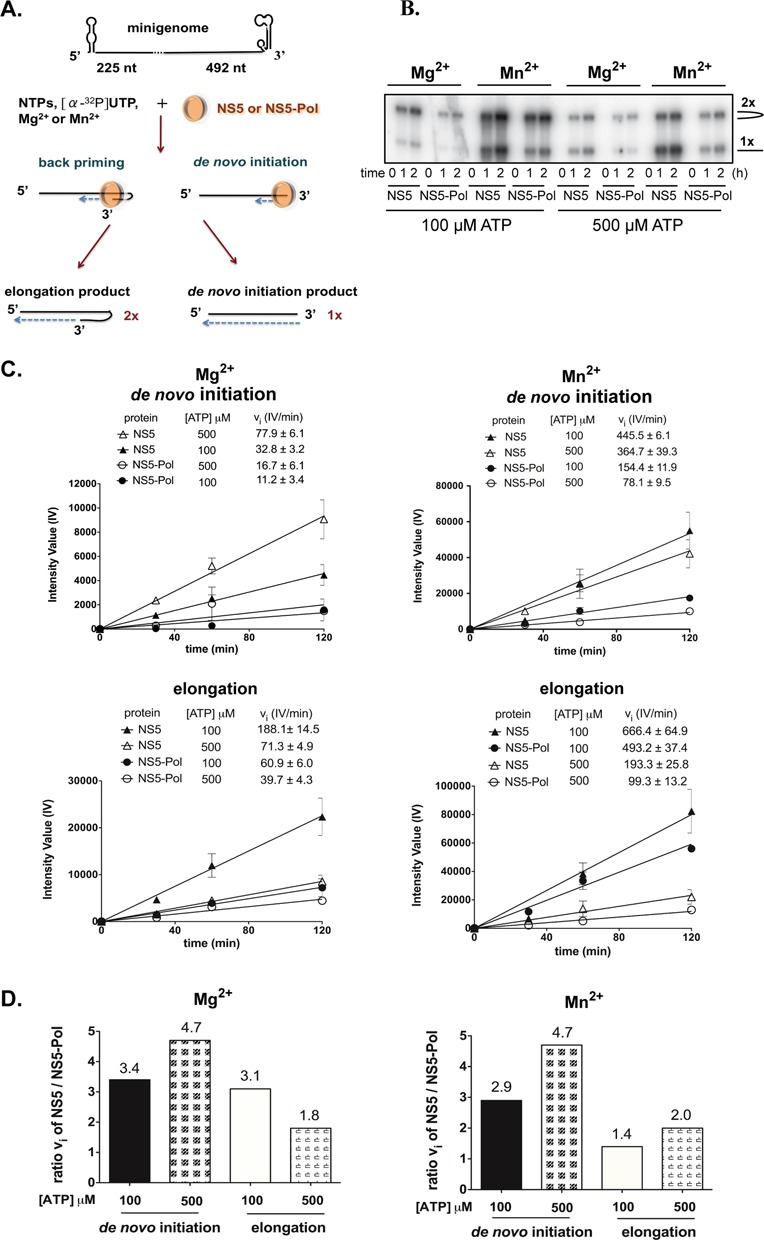
NS5 and NS5-Pol activity on dengue minigenome. (**A**) Schematic presentation of the *in vitro* RdRp assay on a DENV minigenome template. (**B**) Formaldehyde-agarose gel presenting *de novo* initiation and elongation products of NS5 and NS5-Pol (200 nM) with Mg^2+^ or Mn^2+^ and 100 or 500 μM ATP. Reaction conditions are given in Materials and Methods (RdRp assay 2). Reaction kinetics were followed and samples taken after 1 and 2 h. (**C**) Initial velocity graphs of NS5 and NS5-Pol activity showing the generation of *de novo* initiation (upper panels) and elongation (lower panels) products with Mg^2+^ (left) or Mn^2+^ (right) as catalytic ions. Bands on the agarose gels were quantified and intensity values (IV) plotted against time. Initial velocities (*v_i_*) were calculated and are given above the graphs. (**D**) Comparative graphs giving ratios of NS5 to NS5-Pol initial velocities of the generation of *de novo* initiation + elongation and pure elongation products in the presence of Mg^2+^ (left) or Mn^2+^ (right). The reactions were done once and analyzed on agarose gels in duplicates.

### RdRp assay 3: *de novo* initiation tests analyzed by PAGE

Reactions were done at 30°C in HEPES reaction buffer (see RdRp assay 1) containing 2 mM MnCl_2_, or 5 mM MgCl_2_ using NS5 or NS5-Pol at a concentration between 100 nM and 2.5 μM as indicated in the figure legends, either in the absence of template or in the presence 10 μM RNA template (T_10_ or T_20_ indicated in figure legends), and NTPs at concentrations indicated in the figure legends containing either 0.4 μCi/μl [α-^32^P]-GTP or 0.4 μCi/μl [γ-^32^P]-ATP equally indicated in the figure legends. Reactions were started by adding a premix of MnCl_2_, or MgCl_2_, and KCl in HEPES buffer. At given time points, samples were taken and added to equal volumes of formamide/EDTA gel-loading buffer. Reaction products were analyzed using polyacrylamide sequencing gels of 20% acrylamide (19:1), 8 M Urea with TTE buffer (89 mM Tris pH 8.0, 28 mM taurine (2-aminoethanesulfonic acid), 0.5 mM EDTA). RNA product bands were visualized using photo-stimulated plates and the fluorescent Image Analyzer FLA3000 (Fuji) and quantified using Image Gauge (Fuji).

### RdRp assay 4: primer-dependent elongation tests analyzed using PAGE

The 5′-end labeling reaction of primer P_10_ was done with [γ^-32^P] ATP (PerkinElmer) and T4 polynucleotide kinase (New England Biolabs) according to commercial protocols. For primer/template annealing, the 5′-end radiolabeled P_10_ was mixed with T_20_ at molar ratio of 1:1.5, incubated at 70°C for 10 min, cooled down slowly to room temperature and then incubated overnight at 4°C.

Reactions were done at conditions optimized for this specific elongation assay changing reaction temperature to 37°C and using sodium glutamate reaction buffer (50 mM sodium glutamate pH 7.5, 15% glycerol, 5 mM DTT, 15 mM NaCl). Reaction mixtures contained 5 mM MgCl_2_ or 2 mM MnCl_2_, 10 μM P_10_/T_20_ as well as 100 nM protein in the presence of 5 mM MgCl_2_ or 500 nM protein in the presence of 2 mM MnCl_2_. The reactions were started by adding CTP in the reaction buffer to a final concentration of 400 μM. Samples were taken at given time points; and reactions were stopped by adding double volume of formamide/EDTA gel loading buffer. Reaction products were analyzed and visualized as described above for RdRp *de novo* initiation tests.

### Determination of steady-state catalytic parameters

Initial velocity (*v_i_*) values upon steady-state conditions were calculated as the product (P) formation with time during the linear phase of product formation: *v_i_* = *d* [P]/*dt.* Data sets obtained by the determination of *v_i_* values with increasing substrate concentrations were first fitted by non-linear regression to the Hill equation: *v_i_* = *V*_max_[NTP]^h^/((*K*_0.5_)^h^+[NTP]^h^). Data were found to fit best to the classic Michaelis–Menten equation where the Hill coefficient is 1: *v_i_* = *V*_max_ [NTP]/(*K_M_* + [NTP]); *K_M_* = Michaelis constant, *V*_max_ = maximum velocity at infinite substrate concentration. The catalytic constant *k*_cat_ is equivalent to *V*_max_/[protein]. Catalytic efficiency corresponds to *k*_cat_/*K_M_*. The catalytic parameters of NS5 and NS5-Pol were calculated from the equations above by using the GraphPad Prism software.

## RESULTS

### The RdRp activity of NS5 is higher than the activity of NS5-Pol

Recombinant full-length NS5 (residues 1 to 900) and NS5-Pol (residues 272 to 900) of DENV serotype 2 (strain New Guinea C) were expressed in *E. coli* and purified (Supplementary Figure S1A). We first compared the RdRp activities of NS5 and NS5-Pol by filter-binding assays on homopolymeric RNA templates, poly(rC) and poly(rU), and a heteropolymeric minigenome template of about 700 nucleotides (nt) consisting of a fusion of the 5′-end to the 3′-end sequence of the dengue genome (18,31). These filter-binding assays detect the incorporation of [^3^H]-labeled nucleotides into long RNA products generated by *de novo* initiation, transition and elongation (18). Figure 1A–C show the linear part of measured reaction time courses with the calculated initial velocity values given above the graphs. The ratios of initial velocities of NS5 and NS5-Pol (Figure 1D) show that the activity of NS5 is consistently higher, between 6- to 17-fold, than that of NS5-Pol. This indicates that the presence of the NS5-MTase domain stimulates polymerase activity. We then set out to differentiate between the stimulatory effects of the NS5-MTase domain on *de novo* initiation, transition and elongation. In a first approach, we analyzed the products of the minigenome test on denaturing agarose-formaldehyde gels to evaluate possible differences in the stimulation of *de novo* initiation and elongation.

### The presence of NS5-MTase leads to a higher stimulatory effect on *de novo* initiation than on elongation

DENV polymerase on the minigenome template produces a mixture of two products (Figure 2A). One product (here named *de novo* initiation product or 1x product) has template size and is generated by *de novo* initiation over the 3′-end of the template followed by the transition step and elongation of the nascent chain. The second product (elongation or 2x product) is of almost twice the template size and is exclusively formed by elongation promoted by back-priming (17,20,32). Thus by incorporating [^32^P]-labeled UMP and quantitating both products after agarose-formaldehyde gel separation, it is possible to determine whether the NS5-MTase domain preferentially stimulates the *de novo* initiation/transition steps or the elongation phase of RNA synthesis. Polymerization reactions were carried out at two concentrations of the priming nucleotide ATP (100 and 500 μM) since *de novo* initiation is enhanced by higher concentrations of ATP (20). In addition, we used catalytic Mn^2+^ and Mg^2+^ ions separately to compare their influence on the stimulation by the NS5-MTase domain. Figure 2B shows reaction time courses with separated *de novo* initiation products and back-primed elongation products. Figure 2C then presents the quantified intensity values of the *de novo* initiation products (upper panels) and elongation products (lower panels) are then plotted against time for each condition (Mg^2+^ left panels, Mn^2+^ right panels) with the deduced initial velocities shown above the graphs. Analyzing the ratio of NS5 versus NS5-Pol activity (Figure 2D), we observe that NS5 is again consistently more active than NS5-Pol, both in the presence of Mg^2+^ and Mn^2+^. The ratios between NS5 and NS5-Pol activity are higher for products involving *de novo* initiation and transition than for the back-primed elongation products. This is especially true at the higher ATP concentration (500 μM), which is known to stimulate *de novo* initiation. In contrast, the nature of the catalytic ion, Mg^2+^ or Mn^2+^, does not influence the preferential NS5-MTase stimulation during the initiation/transition step. These results together with the results shown in Figure 1 suggest that NS5-MTase domain stimulates *de novo* initiation (possibly the following transition phase) as well as the elongation phase. The pronounced effect upon higher priming ATP concentrations points to a stronger effect of NS5-MTase on *de novo* initiation (with transition) than on elongation. In order to decipher in more detail the stimulation of the *de novo* initiation step by NS5-MTase, we next made use of an assay with short dengue-specific oligonucleotides as templates.

### The NS5-mediated *de novo* initiation activity is higher than that of NS5-Pol

**Figure 3.**
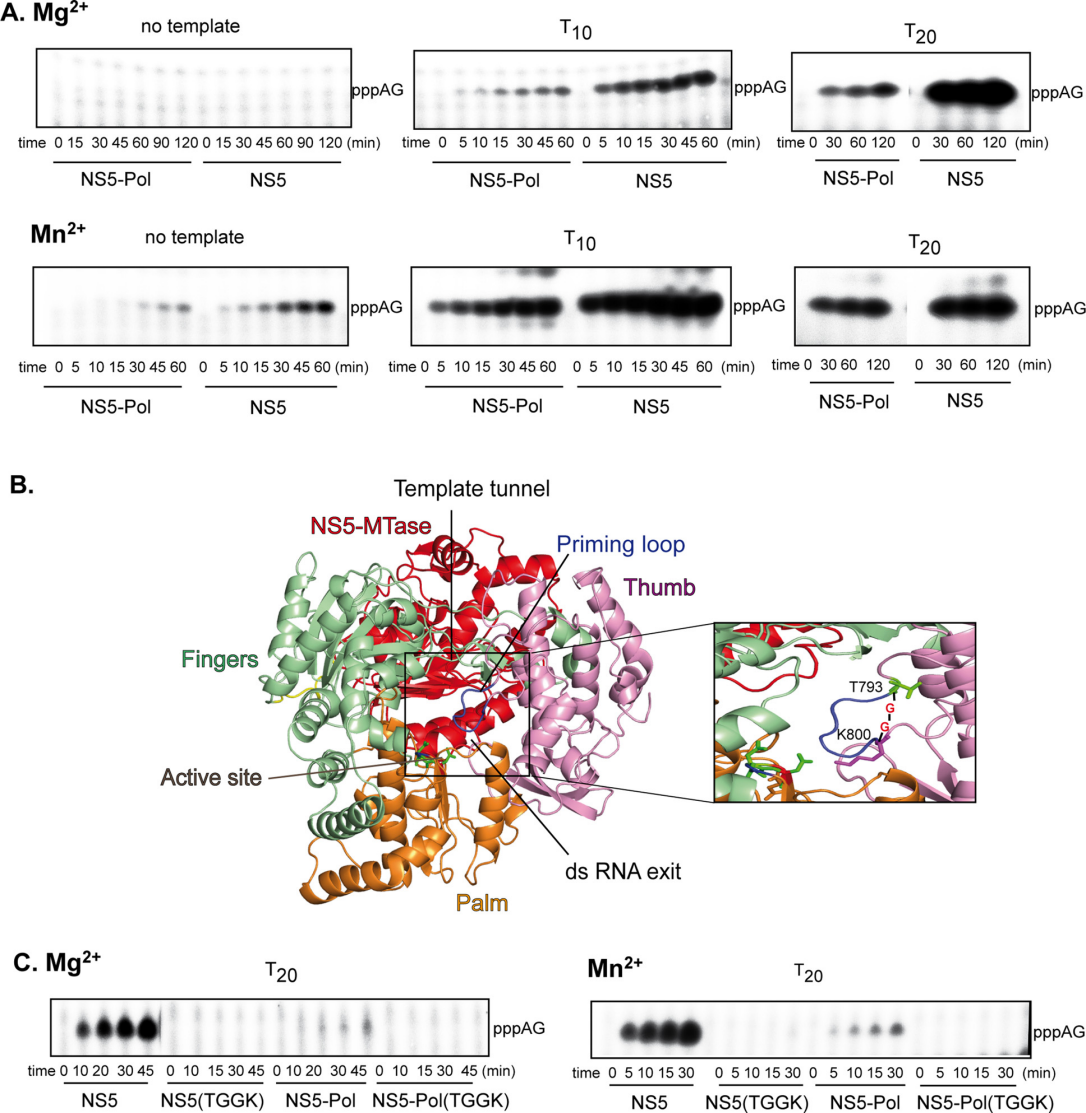
*Specific de novo* initiation activity of NS5 and NS5-Pol. (**A**) and (**C**) *De novo* initiation reactions leading to pppAG formation. The reactions were done as given in Material and Methods (RdRp assay 3) with 500 μM ATP, 100 μM GTP and (A) in the absence of template or with 10 μM T_10_ or T_20_ and 2.5 μM NS5 or NS5-Pol in the presence of 5 mM MgCl_2_ or 0.5 μM NS5 or NS5-Pol in the presence of 2 mM MnCl_2_, or (C) 2.5 μM protein (NS5, NS5(TGGK), NS5-Pol or NS5-Pol(TGGK)) in the presence of 5 mM MgCl_2_ (on the left) or 0.5 μM protein in the presence of 2 mM MnCl_2_ (on the right) and 10 μM T_20_. Radiolabeled GTP (αGTP) was used throughout. Reaction products were analyzed using PAGE. (**B**) A 3D-structural model of DENV 2 NS5 was generated using the structure of JEV NS5 (PDB code 4K6M) and SWISS-MODEL (33). NS5 consists of NS5-MTase (red) and NS5-Pol that adopts the typical closed right-hand structure of viral RdRps containing the palm (orange), fingers (light green) and thumb (light pink) subdomains. The RNA template tunnel entry is situated on the ‘top’ of NS5-Pol between the thumb and fingers subdomains, it leads to the active site harbored mainly by the palm subdomain. The active site contains three conserved aspartic acid residues D533 (motif A) and D663 and D664 (motif C), which are shown in sticks. The NTP tunnel is situated in the ‘back’ of NS5-Pol below NS5-MTase. The priming loop (blue) is part of the thumb subdomain and closes the active site and the dsRNA exit tunnel at the ‘front’. The close-up shows the priming loop T794 to A799. The mutant TGGK of NS5 and NS5-Pol are deletion mutants of this priming loop. The priming loop was replaced by two glycines situated between T793 and K800 (in sticks).

We have shown previously that NS5-Pol produces a short primer, pppAG, on a 10-nt oligonucleotide template (T_10_) corresponding to the 3′-end of the antigenome in the presence of Mg^2+^ and Mn^2+^ (20). In the presence of Mn^2+^ NS5-Pol generates pppAG also in the absence of the template. The short dinucleotide primer is elongated on the template (20). The formation of pppAG thus corresponds to the very *de novo* initiation step. In order to see if the presence of the NS5-MTase domain modifies the *de novo* initiation, we compared the kinetics of pppAG formation by NS5 and NS5-Pol in the absence of a template, on T_10_ and a longer antigenomic template, T_20_, equally with 5′-CU-3′ at its 3′-end, using ATP and GTP (with [α-^32^P]-GTP as a radioactive tracer) in the presence of Mg^2+^ and Mn^2+^. Product formation was followed over time and analyzed using PAGE. As previously observed for NS5-Pol, NS5 is also able to form pppAG in the absence of a template using Mn^2+^ as catalytic ion (Figure 3A, left, lower panel), but not in the presence of Mg^2+^ (left, upper panel). Both proteins generated pppAG using the T_10_ and T_20_ templates in the presence of Mg^2+^ (Figure 3A, upper middle and right panels) and Mn^2+^ (lower middle and right panels). In all these *de novo* reactions, NS5 and NS5-Pol show different efficiencies. The reaction products of NS5 and NS5-Pol for each condition were quantified and the initial velocity values were deduced. Table 1 shows that the specific *de novo* initiation activity of NS5 is around 14-fold higher than that of NS5-Pol in the presence of Mg^2+^, and around 6-fold in the presence of Mn^2+^. We then tested if the apparent stimulation by NS5-MTase could be achieved by adding recombinant NS5-MTase domain in trans to NS5-Pol. Using 0.5 and 2.5 μM NS5-Pol with 0.05 to 5 μM NS5-MTase, we could not detect stimulation (Supplementary Figure S2). In conclusion, we find that NS5-MTase domain strongly stimulates *de novo* initiation confirming the experiments performed with the minigenome template. Either the presence of the cognate linker or simply covalent linking of both domains is essential for the stimulation of the NS5-Pol *de novo* activity by NS5-MTase. In the next step we determined the role played by the priming loop upon *de novo* initiation in the presence of the NS5-MTase domain.

**Table 1.** Comparison of initial velocities of pppAG formation (*de novo* initiation) by NS5 and NS5-Pol in the presence of Mg^2+^ or Mn^2+^ ions

Protein	Divalent ion	Template	*v_i_* (IV/min)	Ratio *v_i_* NS5/NS5-Pol
NS5	Mg^2+^	No template	No formation	-
NS5-Pol	Mg^2+^	No template	No formation	-
NS5	Mg^2+^	T_10_	62.7	10.0
NS5-Pol	Mg^2+^	T_10_	6.3	
NS5	Mg^2+^	T_20_	547.2	17.8
NS5-Pol	Mg^2+^	T_20_	30.8	
NS5	Mn^2+^	No template	93.9	3.9
NS5-Pol	Mn^2+^	No template	24.1	
NS5	Mn^2+^	T_10_	2210.0	5.4
NS5-Pol	Mn^2+^	T_10_	408.0	
NS5	Mn^2+^	T_20_	1639.0	7.5
NS5-Pol	Mn^2+^	T_20_	219.9	

Reactions were done and analyzed as given in Figure 3A. pppAG product bands of reaction time courses were quantified. Initial velocity values are measured through band intensity value per min (IV/min), and are used to compare NS5 over NS5-Pol.

### The priming loop is the major determinant for *de novo* initiation even in the presence of NS5-MTase

We have previously shown that the priming loop of NS5-Pol is crucial for *de novo* initiation (20). In order to test if this is also the case in the context of full-length NS5, deletion mutant NS5(TGGK) was produced, where residues T794 to A799 were replaced by two glycines (Figure 3B). Before comparing its capacity of pppAG formation to wild-type NS5, NS5-Pol and NS5-Pol(TGGK) (20), the proper folding of NS5(TGGK) was assessed by a fluorescent thermal shift assay. NS5(TGGK) shows a temperature of denaturation (melting temperature *T_m_*) similar to that of wild type (see Supplementary Figure S1B). The *T_m_* values of NS5 proteins are 2°C lower than those of the NS5-Pol proteins. This moderate stability difference is also found testing chemical denaturation by guanidine hydrochloride (see Supplementary Figure S1C). As an activity control of the proper folding of the mutant proteins we measured the elongation activity of NS5(TGGK) and NS5-Pol(TGGK), which is not affected by the deletion of the priming loop (Supplementary Figure S3). We then compared the *de novo* initiation properties of the wild-type and mutant proteins by measuring their abilities to synthetize pppAG (Figure 3C). In contrast to wild-type proteins, both mutant proteins NS5(TGGK) and NS5-Pol(TGGK) do not support *de novo* initiation. In conclusion, our results demonstrate that the priming loop remains the major determinant required for *de novo* initiation in the context of full-length NS5. In the next step we investigated how NS5-MTase stimulates *de novo* initiation by comparing the affinities of NS5-Pol and NS5 to the RNA template and the priming ATP.

### NS5 has a higher affinity than NS5-Pol for both the single-strand RNA template and the priming ATP

**Figure 4.**
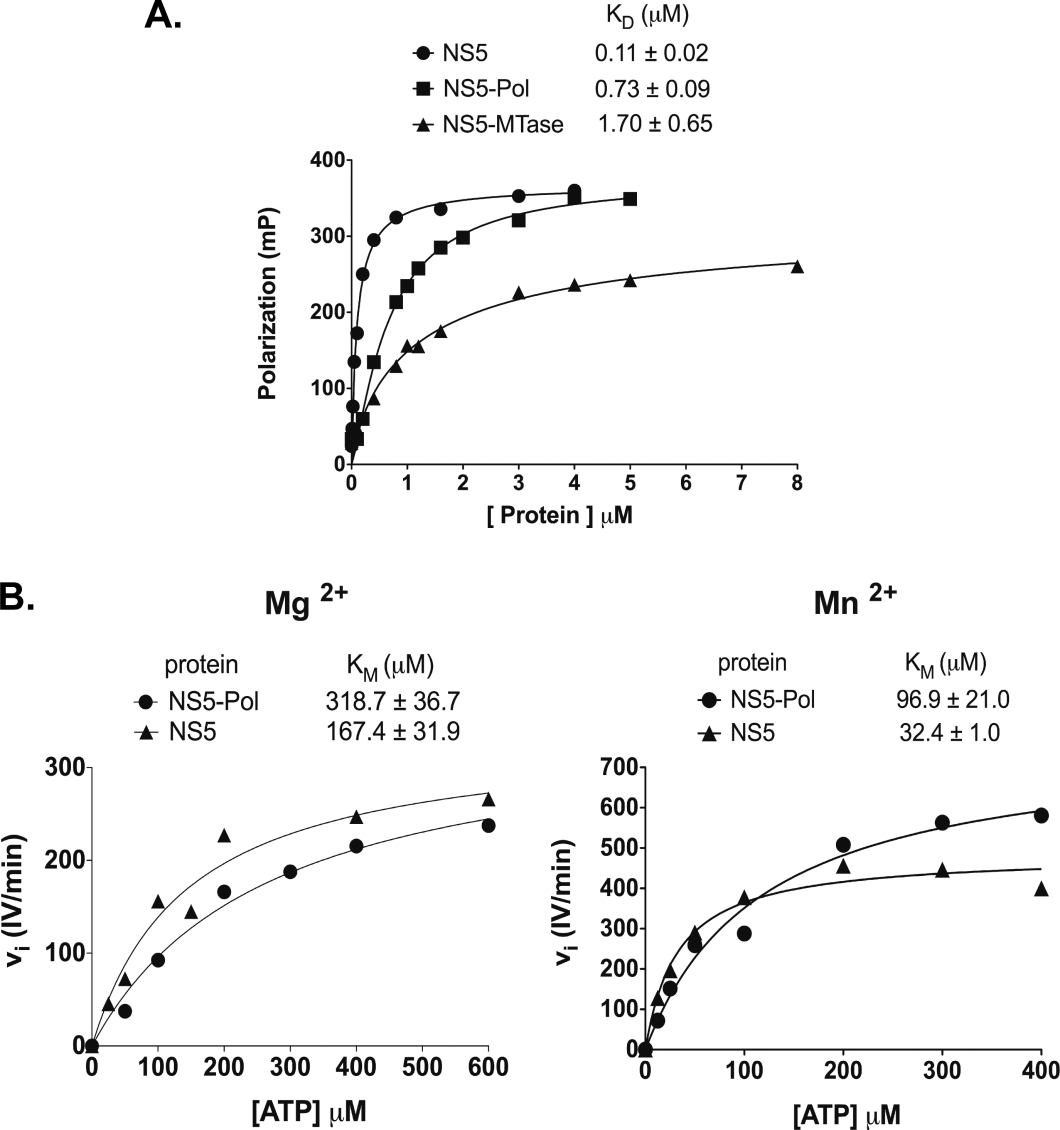
NS5 and NS5-Pol RNA template affinity and *K_M_* values of the priming nucleotide during *de novo* initiation. (**A**) Dissociation constants (*K_D_*) of NS5 and NS5-Pol from fluorescence polarization tests. Binding assay was performed with increasing concentration of proteins as given in Materials and Methods and 10 nM T_20_ template bearing 6-FAM at the 5′-end. The experiments were done in triplicates. The *K_D_* values of NS5, NS5-Pol and NS5-MTase were determined by using a specific one-site binding equation. The curves correspond to one test. The *K_D_* values above the graph represent average *K_D_* values of three independent tests. (**B**) ATP *K_M_* values of NS5 and NS5-Pol are given above the graphs plotting initial velocities of 0.5 μM NS5 or NS5-Pol at ATP concentrations of 25 to 800 μM in the presence of 5 mM MgCl_2_ (on the left), or of 0.1 μM NS5 or NS5-Pol, at ATP concentrations of 12.5 to 400 μM in the presence of 2 mM MnCl_2_ (on the right). Reactions were performed as given in Material and Methods (RdRp assay 3). In both cases 10 μM T_20_ template and 100 μM GTP was used. The data sets can be described by a classic Michaelis–Menten equation (Hill slope set to 1, see Materials and Methods). The curves correspond to one of three independent tests. The given *K_M_* values above the graphs correspond to an average *K_M_* value.

The *de novo* initiation of RNA synthesis depends on the RNA template being loaded into the RNA entry tunnel with the 3′-end reaching the active site. Single-strand RNA binding properties of NS5 and NS5-Pol were measured using a fluorescent polarization (FP) assay. For this purpose, RNA template T_20_ bearing a fluorescent marker at the 5′-end, was incubated with increasing concentrations of either NS5 or NS5-Pol in reaction buffer containing Mg^2+^ ions, and the change in FP was measured (Figure 4A). The affinity constant *K_D_* deduced for NS5 is 0.11 μM, i.e. 6.6-fold lower than that of NS5-Pol (0.73 μM). The *K_D_* value of the NS5-MTase domain, used as a control, is 1.7 μM. These results suggest that a higher affinity of NS5 toward a single-strand RNA in comparison to NS5-Pol contributes to the enhanced *de novo* initiation activity of NS5. This increase in affinity is not related to a high-affinity binding site on the NS5-MTase domain as indicated by its low *K_D_* value.

We next addressed the possibility that the NS5-MTase domain influenced the *de novo* initiation step by lowering the affinity of the priming nucleotide ATP. Note that is has been shown that the NS5-MTase domain is not able to bind ATP by itself (34). We determined the ATP *K_M_* values, by measuring the initial velocities of pppAG formation at increasing ATP concentrations (Figure 4B). The *K_M_* values of the priming ATP are 318.7 μM for NS5-Pol and 167.4 μM for NS5 in the presence of Mg^2+^. They are significantly lower in the presence of Mn^2+^: 96.9 μM for NS5-Pol and 32.4 μM for NS5. Thus, the *K_M_* values of NS5-Pol are 1.9- and 3.0-fold higher than those of NS5 in the presence of Mg^2+^ and Mn^2+^, respectively. Altogether our results indicate that the NS5-MTase domain stimulates the initiation step by increasing both the RNA template recruitment and by increasing the affinity of ATP to the priming site. We next addressed the influence of the NS5-MTase domain on the transition phase of RNA synthesis.

### NS5 and NS5-Pol show a rate-limiting transition phase between initiation and elongation

**Figure 5.**
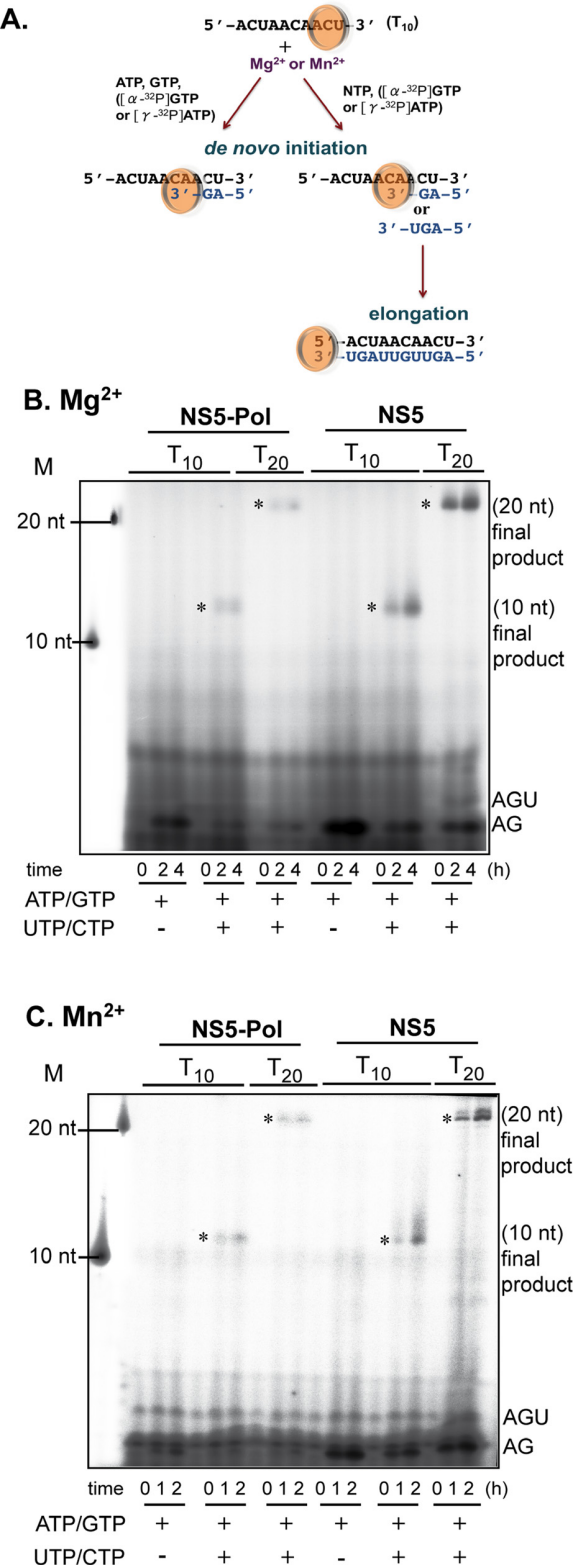
*De novo* initiation, transition and elongation by NS5 and NS5-Pol on short dengue-specific templates. (**A**) Schematic presentation of *in vitro* RdRp *de novo* initiation and elongation tests on dengue antigenomic T_10_ and T_20_ templates. (**B**) The reactions were done as given in Materials and Methods (RdRp assay 3). Reaction kinetics were followed and samples analyzed using PAGE. Reaction mixtures contained 0.5 μM NS5 or NS5-Pol, 10 μM T_10_ or T_20_ templates, 500 μM ATP, 100 μM GTP and the given concentration of UTP and CTP, 5 mM MgCl_2_ and [α-^32^P]-GTP. M stands for marker oligos corresponding to the labeled templates. Identities of products are given on the right. (**C**) As in (B) but reactions contained 0.5 μM NS5 or NS5-Pol, 1 μM T_10_ or T_20_ templates, 100 μM ATP, 500 μM GTP, the given concentration of UTP and CTP, 2 mM MnCl_2_ and [γ-^32^P]-ATP. Full-length products (labeled by asterisks) migrate slightly higher than the labeled templates used as size marker since they bear a triphosphate moiety at their 5′-end and their sequence is complementary.

When all four nucleotides are used on the antigenomic template T_10_, NS5-Pol was shown to accumulate short primers before entering into a processive elongation mode (20). This accumulation indicates a transition phase, which is rate-limiting under these test conditions, and might correspond to the necessary conformational change between initiation and elongation. In order to see if the presence of the NS5-MTase domain modifies the apparent rate-limiting nature of the transition, we tested NS5 in comparison to NS5-Pol in the presence of Mg^2+^ and Mn^2+^. Either the specific *de novo* initiation product pppAG was synthesized using ATP and GTP in control reactions; or complete reactions were set up using all four NTPs (Figure 5A). Product formation was followed over time and analyzed using PAGE (Figure 5B (Mg^2+^) and 3C (Mn^2+^)). On both templates and with both catalytic ions, NS5 and NS5-Pol generate similar product profiles. They accumulate short primers, pppAG and to a lesser extent pppAGU, before entering into a processive elongation mode generating full-length products (labeled by asterisks). These results indicate that the presence of the NS5-MTase domain does not abolish the apparent rate-limiting nature of the transition phase. We then analyzed in the final part the effect of the NS5-MTase domain on elongation.

### NS5 shows a higher activity than NS5-Pol during elongation

**Figure 6.**
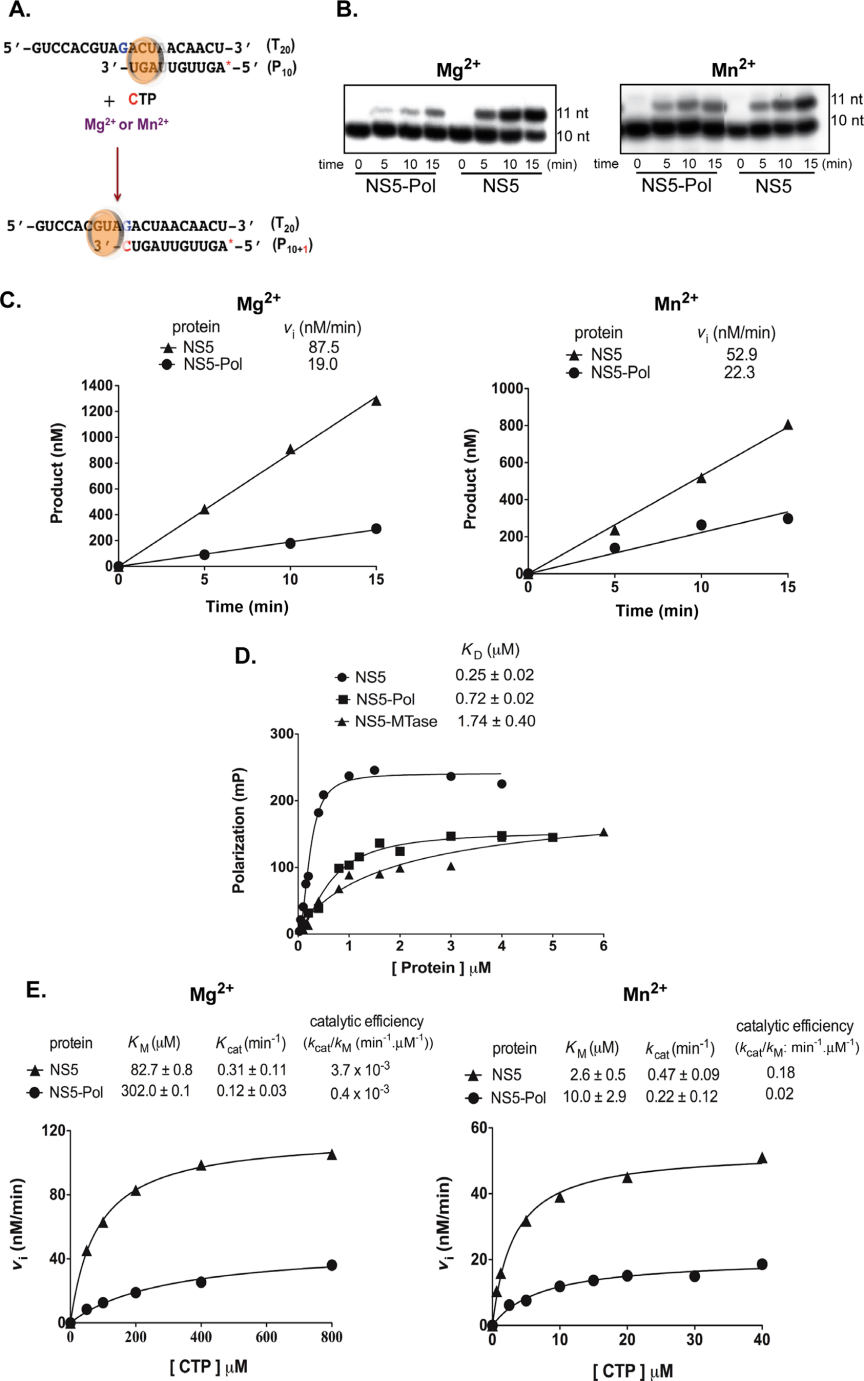
Primer-dependent elongation activity of NS5 and NS5-Pol. (**A**) Reaction scheme showing the primer/template where a single CTP is incorporated. The red star represents the radioactive label at the 5′-end of the primer. (**B**) Reaction time courses for elongation reactions by NS5 and NS5-Pol (see Material and Methods RdRp assay 4). Samples were taken at given time points and analyzed by PAGE. Primer (10 nt) and elongation products (11 nt) are shown. (**C**) Graphs show quantified reaction products from (B) plotted against time. Resulting *v_i_* values are given above the graphs. (**D**) *K_D_* of NS5 and NS5-Pol from fluorescence polarization tests. Binding assay was performed with 8 nM P_10_/T_20_ and increasing concentrations of proteins as given in Material and Methods. The experiments were done in triplicates. The curves correspond to one representative test. The *K_D_* values shown above the graphs are average *K_D_* values. (**E**) Catalytic parameters of NS5 and NS5-Pol during elongation. Initial velocities were determined with 0.5 μM NS5 or NS5-Pol and increasing concentrations of CTP (25 μM to 1 mM) in the presence of 5 mM MgCl_2_, or 100 nM NS5 or NS5-Pol, 12.5 to 40 μM CTP in the presence of 2 mM MnCl_2_ always with 10 μM P_10_/T_20_. The data sets can be described by a classic Michaelis–Menten equation (Hill slope set to 1, see Materials and Methods). The curves correspond to one of three independent tests. The given catalytic parameters are given above the graphs and correspond to average values.

In order to specifically follow elongation, we measured single-nucleotide incorporation into a specific primer/template. The dengue antigenomic T_20_ template annealed to a complementary radiolabeled primer of 10 nt (P_10_) was incubated with NS5 and NS5-Pol in the presence of Mg^2+^ or Mn^2+^. The next nucleotide (CTP) was added (Figure 6A) and reaction mixtures were analyzed using PAGE (Figure 6B, Mg^2+^ on the left and Mn^2+^ on the right). Two main bands, corresponding to the labeled primer (10 nt) and to the elongation product (11 nt) were detected on the gel. Figure 6 shows that both NS5 and NS5-Pol use P_10_/T_20_ as a substrate. Both proteins are able to incorporate CTP into the primer. The reaction products were quantified, plotted against time (Figure 6C) and initial velocities determined. They are shown above the graphs. Comparing initial velocities of NS5 and NS5-Pol, we observe that NS5 is 4.6- and 2.3-fold more active than NS5-Pol in the presence of Mg^2+^ and Mn^2+^, respectively. In conclusion, as suggested by the minigenome assay (Figure 2), the NS5-MTase domain also provides stimulation of the elongation phase, but this stimulation is less important than that observed for *de novo* initiation (Table 1). As for the initiation step we then asked if the activity difference seen in the elongation test is due to a better primer/template binding and/or a change in the catalytic constants of NS5-Pol.

### NS5 exhibits both a higher affinity than NS5-Pol toward primer-template RNA for elongation and a higher catalytic efficiency

The affinities of NS5 and NS5-Pol to a primer/template RNA were measured using FP. Increasing concentrations of NS5 and NS5-Pol were incubated with P_10_/T_20_ bearing a fluorescent marker at the 5′-end of the primer. The affinity value of P_10_/T_20_ toward NS5-MTase was measured as a control. Figure 6D shows that NS5 has a dissociation constant *K_D_* of 0.25 μM, i.e. 2.9-fold lower than that of NS5-Pol (0.72 μM). The corresponding *K_D_* value for NS5-MTase (1.74 μM) is about 7-fold higher than that of NS5. The presence of NS5-MTase increases thus the affinity of NS5 toward a RNA primer/template contributing to the difference between NS5 and NS5-Pol in the specific elongation activity test. In order to compare catalytic efficiencies of NS5 and NS5-Pol upon elongation, initial velocities were measured varying the concentration of CTP in the presence of Mg^2+^ or Mn^2+^ (Figure 6E, Mg^2+^ on the left and Mn^2+^ on the right). *K_M_* values (shown above the graphs) of CTP upon elongation by NS5 are 82.7 μM in the presence of Mg^2+^ and 2.6 μM in the presence of Mn^2+^. The corresponding *K_M_* values of NS5-Pol are 4-fold higher than those of NS5. The *k*_cat_ values for NS5 are around 2-fold higher than those of NS5-Pol irrespective of the catalytic ions. Consequently, the catalytic efficiency value corresponding to *k*_cat_/*K_M_* of NS5 is 9-fold higher than that of NS5-Pol upon elongation. Altogether, we conclude that the NS5-MTase domain stimulates elongation by facilitating RNA recruitment, by increasing both the affinity of the catalytic site to the incoming nucleotide and the catalytic constant of nucleotide incorporation.

## DISCUSSION

We have used an array of steady-state experiments to compare the RdRp activity of full-length DENV-2 NS5 with that of its isolated polymerase domain NS5-Pol. We find that NS5 and NS5-Pol follow the same three steps RNA synthesis scheme involving: (i) *de novo* generation and accumulation of short primers for which the priming loop is a major determinant, (ii) a transition phase consisting in a conformational change of the NS5-Pol domain from the initiation to the elongation conformation and (iii) compressive elongation. The NS5-MTase domain, within the context of full-length NS5, is actively supporting the polymerase activity carried by the NS5-Pol domain. NS5 presents consistently higher initial velocities in comparison to NS5-Pol in the presence of either Mg^2+^ or Mn^2+^ as catalytic ion. We have also found that the presence of S-adenosyl methionine (SAM), the co-substrate of the NS5-MTase during methylation, does not change the RdRp activity of NS5 nor do the mutations of the essential NS5-MTase active site residue D146 or the essential cap-binding site residue F25 to alanine (data not shown). The stimulation occurs during both the *de novo* initiation phase and the elongation phase. Two observations lead us to propose that the NS5-MTase domain enhance *de novo* initiation to a higher extent than elongation. First, using the minigenome assay we observe more important differences between NS5 and NS5-Pol regarding the amount of a product generated by initiation/transition and elongation versus the amount of a product generated exclusively by elongation (Figure 2). These differences are especially high at a higher priming ATP concentration when more initiation events occur. Second, differences in initial velocities are considerable higher upon *de novo* initiation (Table 1) in comparison to elongation (Figure 6).

The mechanistic basis for the observed stimulation was addressed by measuring RNA affinity values and steady-state catalytic constants of NS5 and NS5-Pol using specific *de novo* initiation and elongation tests. First, we measured the affinity of NS5 toward the ssRNA template used in the *de novo* initiation test independently from RNA synthesis. The *K_D_* value of NS5 was around 7-fold lower than the *K_D_* value of NS5-Pol (see summary in Table 2). The higher affinity of NS5 toward the ssRNA points to the importance of the NS5-MTase domain for RNA template loading. In the context of full-length NS5, the NS5-MTase domain could either extend the RNA binding surface creating a larger binding site with a lower overall *K_D_* value, and/or promote the proper conformation of the RNA template tunnel changing the affinity of the NS5-Pol RNA binding region. In contrast, NS5 showed only a 3-fold higher affinity for primer/template RNA than NS5-Pol. The affinity difference between NS5 and NS5-Pol toward a primer/template could be relevant if the transition phase proceeds via dissociation, change of conformation and re-association of the polymerase to a primer/template before engaging into processive elongation. Concerning the catalytic constants, we measured *K_M_* values for the initiating nucleotide ATP, which binds to the priming site upon pppAG formation, and for the incoming nucleotide, which binds to the catalytic site upon elongation. We find that the presence of the NS5-MTase domain significantly decreases both *K_M_* values (2- to 4-fold, see Table 2). Our results are in accordance with observed differences in NTP *K_M_* values of DENV NS5 and NS5-Pol using more complex reactions including initiation/transition and elongation (see Table 2, (18,35)). Finally, we show evidence that the stimulation provided by the NS5-MTase domain translates into a 2- to 3-fold increase in the catalytic rate constant value of NTP incorporation during elongation. In conclusion, the mechanistic basis of the observed support of the *de novo* initiation and elongation steps of RNA synthesis by the NS5-MTase domain consists, at least in part, of (i) a stimulation of RNA template loading, (ii) an increase in the binding affinity of the substrate NTPs to the priming site and the catalytic site and (iii) an increase in the catalytic rate of NTP incorporation during elongation.

**Table 2.**
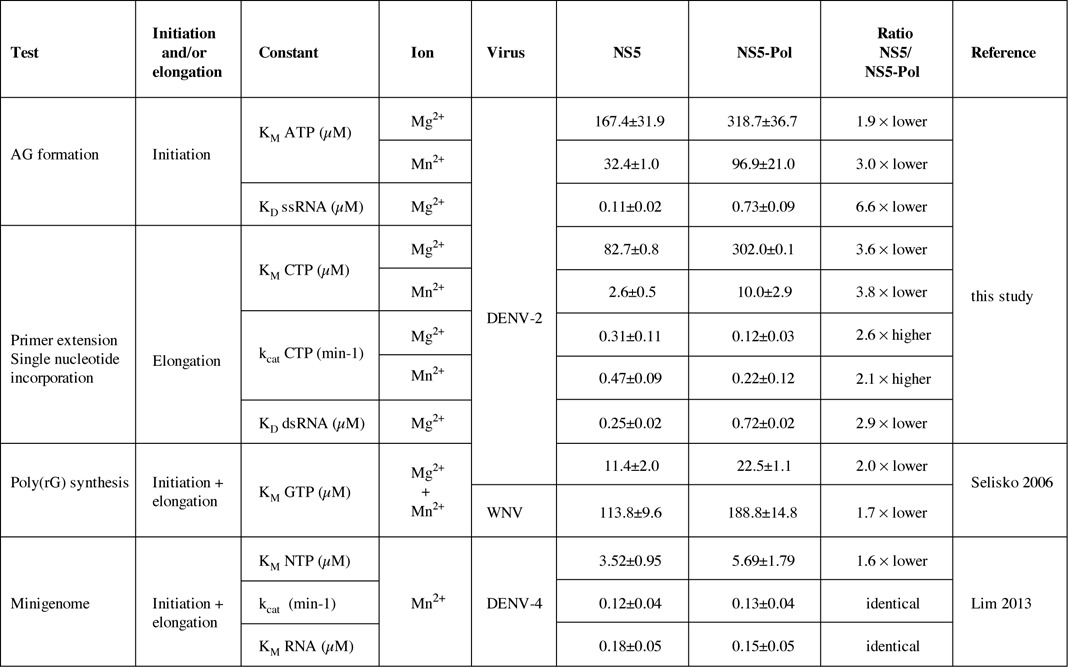
Summary of steady-state kinetic constants determined in this study, by Selisko (18) and Lim (35)

Our work does not directly demonstrate how the methyltransferase domain regulates the activities of the polymerase domain. To answer this question, more biochemical and structural information is needed on wild type and mutant forms of NS5. We need 3D structures of *Flavivirus* NS5 in complex with different RNA substrates, NTPs and divalent ions that show how NS5-MTase and NS5-Pol physically interact, and mutant data on the interface residues to demonstrate if a particular interface indeed regulates the RdRp activity of NS5-Pol. Nevertheless, based on our results and the structural information on conformational changes of RdRp domains during the different steps of RNA synthesis, we expect that the NS5-MTase domain establishes at least one stimulatory interface with the NS5-Pol domain ensuring efficient RNA loading, initiation and elongation. The NS5-MTase domain does not seem to simply support NS5-Pol folding. A comparison of thermal and chemical stability of both proteins measuring melting temperature values and chemical denaturation midpoints (see Supplementary Figure S1) showed that NS5 and NS5-Pol (with and without N-terminal His-tag) follow similar typical denaturation curves of globular proteins. NS5-Pol is moderately more stable than NS5. This is in accordance with a recent study (35) where the stability of DENV-4 NS5 and NS5-Pol (both without a His-tag) were compared and nearly identical *T_m_* values (37.5°C and 38°C, respectively) were found. The fact that we could not re-constitute the stimulatory interface during *de novo* initiation by adding NS5-MTase to NS5-Pol in trans (Supplementary Figure S2) indicates that the physical closeness of the two domains is required for the formation of the *de novo* interface. The inter-domain affinity in the absence of the linker seems to be relatively low. It is also possible that the linker itself is implicated in the interface formation. The importance of the linker was reported by Dong *et al.* (24), who found stimulation of an internal methylation activity of NS5-MTase by the NS5-Pol domain only in the context of full-length NS5. Interestingly, Lim *et al.* (35) added a part of the linker (maximum nine residues) to recombinant DENV NS5-Pol, and observed that *k*_cat_ values measured with a minigenome test increased and even got higher than for full-length NS5, whereas the *K_M_* values for NTPs and RNA did not change. A DENV-3 NS5-Pol structure with the linker showed that stabilization may be caused by an additional salt bridge. The residues providing the salt bridge are conserved in DENVs but not in other flaviviruses. Accordingly, the JEV NS5 structure presents a highly flexible linker. Within the context of full-length DENV NS5, this potential stabilization by the linker is not apparent (this study and (35)).

The multi-step collaboration between the *Flavivirus* NS5-Pol and NS5-MTase domains guarantees a highly efficient synthesis of viral RNA, the detailed understanding of which requires further functional and structural studies of RNA-NS5 complexes corresponding to each step. There are two possible scenarios for the collaboration between both domains: either NS5-MTase exerts its stimulation *via* one flexible interface with NS5-Pol or it establishes several interfaces corresponding to each step of RNA synthesis. Both scenarios may be related to the compact structure of full-length JEV NS5 (14) where the NS5-MTase stabilizes the fingers subdomain of NS5-Pol being situated on the ‘back’ of NS5-Pol behind the RNA template entrance (see Figure 4B). In this structure the NS5-MTase domain is well placed to promote and stabilize the RNA tunnel and could potentially extend the RNA-template binding zone. In this way NS5-MTase would promote RNA loading, i.e. the entry of the minus-strand 3′-end, as we have seen in our functional tests. Optimal RNA loading and tunnel structuration should involve a closure of the distance between fingers and thumb subdomains as observed in *de novo* initiating structures of HCV RdRp (19). In the first scenario, the optimal placement of the RNA would then induce the proper structuring of the priming and catalytic site during initiation and elongation. The NS5-MTase-Pol interface should allow some flexibility to accommodate the conformational changes of NS5-Pol before initiation and upon transition toward elongation. The priming loop has to move out of the way and the polymerase domain has to go back to an open form leaving space for a primer/template. Nascent dsRNA will leave NS5-Pol toward the front of the molecule (see Figure 4B). Thus, for RNA capping the 5′-end of the nascent positive-strand RNA will have to join the NS5-MTase domain at the back of the polymerase or use the NS5-MTase domain of another NS5 protein placed in front. Within the second scenario the NS5-MTase domain would exerts its pleiotropic effect on the NS5-Pol domain activity by a change of its location on the surface of the NS5-Pol domain. The conformation during RNA loading may again correspond to full-length JEV NS5 (14) as discussed above. The proper structuring of the RNA channel including the closure of the distance fingers/thumb would then allow a re-positioning of the NS5-MTase toward the ‘front’ of the polymerase, where the priming loop is situated (see Figure 4B). The linker indeed provides the flexibility and the necessary distance to allow the re-positioning. NS5-MTase could thus, via a direct interaction, stabilize the proper conformation of the priming loop, i.e. increase the affinity of the ATP priming site. After pppAG formation and transition, the processive elongation phase takes place, and according to our results, the NS5-MTase contributes to the proper conformation of the NTP catalytic site and also speed up incorporation. It may act through another, elongation-specific, interface with NS5-Pol. Given the role of the NS5-MTase domain in RNA capping, the domain might stay in the ‘front’ near the dsRNA exit to exert its function at the 5′-end of a nascent positive strand. Future experiments are needed to clarify, which scenario the *Flavivirus* replication complex (RC) adopts.

Within the membrane-bound *Flavivirus* RC, NS5 interacts with NS3 (13,36), which bears the RNA helicase and triphosphatase (first step of RNA capping) functions (reviewed in (37)). NS5 is only indirectly connected to the membrane via NS3, which in turn binds to the membrane-associated viral RC proteins NS2A, NS4A and NS4B (36). Of course, NS5 might also contact host RC proteins; at the moment numerous interaction candidates exist, which have yet to be corroborated (38). We expect that NS3 has an influence on the interaction of NS5-MTase and NS5-Pol, supposedly exerting its enzymatic activities before, during and/or after RNA synthesis and capping. NS3 itself should thus establish one or several interfaces with NS5. Our knowledge on the interaction between NS3 and NS5 is still quite limited. There is evidence that the NS3 helicase domain interacts with NS5-Pol via an interface at the back of the thumb subdomain involving a loop coming from the fingers subdomain and connecting to the thumb (39,40). This would be very close to the NS5-MTase in the JEV NS5 structure (see Figure 3B). It might be there, where NS3 exerts its function as a helicase before or during RNA synthesis. NS3 might adopt a different position when it acts as the RNA triphosphatase during or after +RNA synthesis. It will be interesting to see whether the stimulatory effects of the NS5-MTase on NS5-Pol are influenced by the presence of NS3. We expect that the emerging functional intra- and inter-protein interfaces of the complex NS5-NS3, the heart of the *Flavivirus* RC, will constitute valuable targets for drug discovery and development.

## SUPPLEMENTARY DATA

Supplementary Data are available at NAR Online.

SUPPLEMENTARY DATA
