# The methyltransferase domain of dengue virus protein NS5 ensures efficient RNA synthesis initiation and elongation by the polymerase domain

**DOI:** 10.1093/nar/gkv1294

**Published:** 2015-11-17

**Authors:** Supanee Potisopon, Stéphane Priet, Axelle Collet, Etienne Decroly, Bruno Canard, Barbara Selisko

**Affiliations:** 1 Aix-Marseille Université, AFMB UMR 7257, 13288 Marseille, France; 2 CNRS, AFMB UMR 7257, 13288 Marseille, France

*Nucl. Acids Res*. 42 (18): 11642–11656. doi: 10.1093/nar/gku666

The authors wish to make the following corrections to Figure 1:

**Figure 1.**
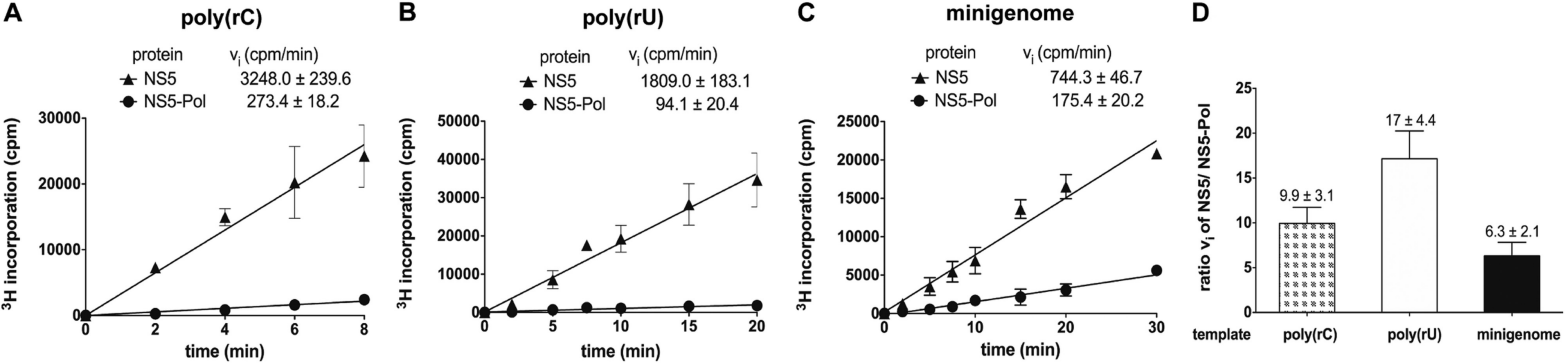
NS5 and NS5-Pol activity on homopolymeric and heteropolymeric templates analyzed by filter binding. Reactions kinetics were determined as given in Materials and Methods (RdRp assay 1). Reactions were done in HEPES reaction buffer with (A) 100 nM poly(rC), 40 nM protein, 20 μM GTP, (B) 100 nM poly(rU), 100 nM protein, 200 μM ATP, (C) 100 nM minigenome, 100 nM protein, 300 μM ATP, CTP, GTP, 4 μM UTP. Incorporation of 3H-labeled NTPs was measured in counts per minute (cpm). (D) Ratios of NS5 and NS5-Pol activities are given for different RNA templates. The tests were done in triplicates.

In the original figure, panels A and B were accidently duplicated. A corrected Figure is provided below.

The results and conclusion of the article are not affected and remain valid. The authors apologise to the readers for the inconvenience caused.

