# Supplementary material for: The methyltransferase domain of dengue virus protein NS5 ensures efficient RNA synthesis initiation and elongation by the polymerase domain
Source: Nucleic Acids Res. 2014 Sep 10;42(18):11642–56. doi: 10.1093/nar/gku666 (PMC4191377; doi:10.1093/nar/gku666)
Supplement: SUPPLEMENTARY DATA [file supp_42_18_11642__index.html]

The methyltransferase domain of dengue virus protein NS5 ensures efficient RNA synthesis initiation and elongation by the polymerase domain — SUPPLEMENTARY DATA 

# The methyltransferase domain of dengue virus protein NS5 ensures efficient RNA synthesis initiation and elongation by the polymerase domain

## SUPPLEMENTARY DATA

**Files in this Data Supplement:**

- SUPPLEMENTARY DATA
