# Supplementary material for: The methyltransferase domain of dengue virus protein NS5 ensures efficient RNA synthesis initiation and elongation by the polymerase domain
Source: Nucleic Acids Res. 2014 Sep 10;42(18):11642–56. doi: 10.1093/nar/gku666 (PMC4191377; doi:10.1093/nar/gku666)
Supplement: SUPPLEMENTARY DATA [file supp_gku666_nar-01226-r-2014-File008.pdf]

SUPPLEMENTARY FIGURES

Figure S1: NS5 and NS5-Pol purity and stability. (A) 12 % SDS-PAGE gel presents recombinant purified NS5 and NS5-Pol proteins and molecular marker proteins (left lane M). The molecular mass of markers is given on the left. **(B)** T_m values of NS5 and NS5-Pol measured by thermo-fluor based assay described in Material and Methods. **C)** Guanidine hydrochloride concentration midpoints of NS5 and NS5-Pol denaturation determined as described in Materials and Methods. The experiments were done at least in triplicates.

Figure S2: Absence of stimulation by NS5-MTase when added as recombinant protein to NS5-Pol

De novo initiation reactions leading to pppAG formation in the absence and presence of increasing concentrations of NS5-MTase. Product formation was followed with time and analyzed using PAGE. The reactions were done as given in Materials and Methods with 500 µM ATP, 100 µM GTP, 10 µM T_{20} and 2 mM $MnCl_{2.}$ NS5 and NS5-Pol were used without His-tag. NS5-MTase concentrations are given below the gel.

Figure S3: Primer-dependent elongation activity of NS5, NS5 (TGGK), NS5-Pol and NS5-Pol (TGGK). Reactions were done and analyzed using PAGE as described in Materials and Methods. **(A)** in the presence of $MgCl_2$, 0.5 µM protein, 10 µM P_{10}/T_{20}, **(B)** in the presence of $MnCl_2$, 0.1 µM protein, 10 µM P_{10}/T_{20}.

Figure S1

A.

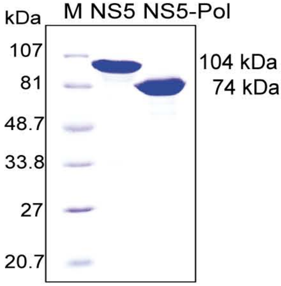

B.

protein	His-tag	T_m (°C)
NS5	+	38.0 ± 0.2
NS5	-	37.1 ± 0.1
NS5(TGGK)	+	37.7 ± 0.1
NS5-Pol	+	40.1 ± 0.2
NS5-Pol	-	40.1 ± 0.1
NS5-Pol(TGGK)	+	39.8 ± 0.0
NS5-MTase	+	41.6 ± 0.0

C.

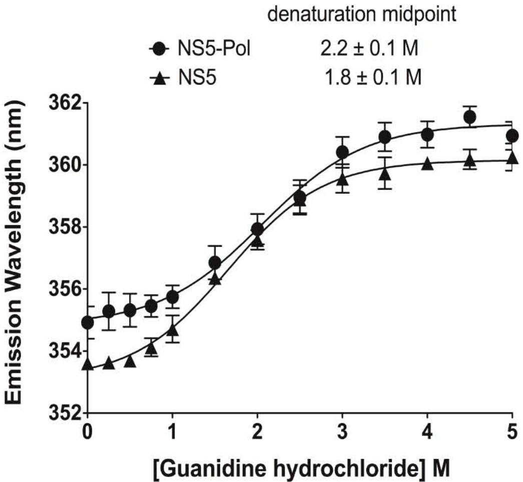

Figure S2

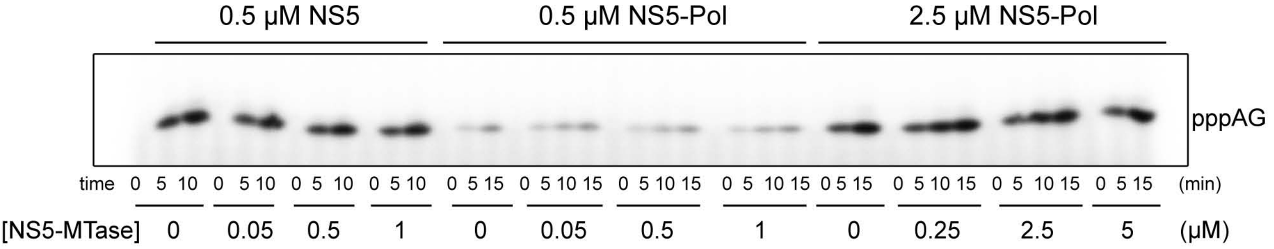
0.5 μM NS5
0.5 μM NS5-Pol
2.5 μM NS5-Pol
pppAG
time 0 5 10 0 5 10 0 5 10 0 5 10 0 5 15 0 5 10 15 0 5 10 15 0 5 10 15 0 5 15 0 5 10 15 0 5 10 15 0 5 15 (min)
[NS5-MTase] 0 0.05 0.5 1 0 0.05 0.5 1 0 0.25 2.5 5 (μM)

Figure S3

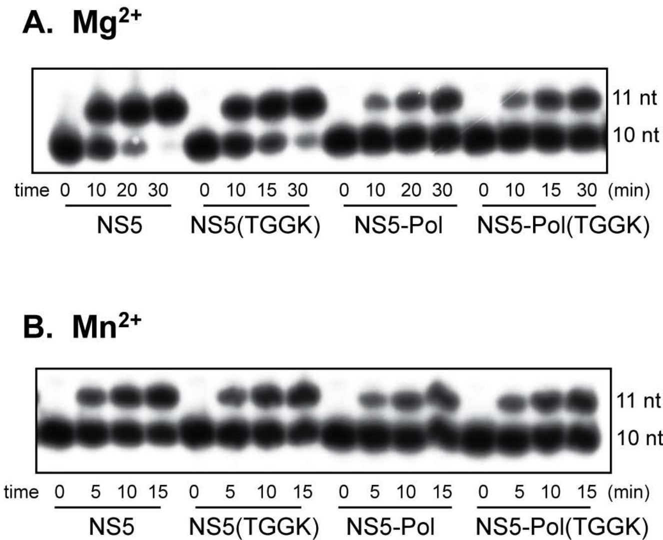
A. Mg^{2+}
11 nt
10 nt
time 0 10 20 30 0 10 15 30 0 10 20 30 0 10 15 30 (min)
NS5
NS5(TGGK)
NS5-Pol
NS5-Pol(TGGK)
B. Mn^{2+}
11 nt
10 nt
time 0 5 10 15 0 5 10 15 0 5 10 15 0 5 10 15 (min)
NS5
NS5(TGGK)
NS5-Pol
NS5-Pol(TGGK)
